# Ultrathin TiO_2_ Coatings via Atomic Layer Deposition Strongly Improve Cellular Interactions on Planar and Nanotubular Biomedical Ti Substrates

**DOI:** 10.1021/acsami.3c17074

**Published:** 2024-01-26

**Authors:** Jan Capek, Marcela Sepúlveda, Jana Bacova, Jhonatan Rodriguez-Pereira, Raul Zazpe, Veronika Cicmancova, Pavlina Nyvltova, Jiri Handl, Petr Knotek, Kaushik Baishya, Hanna Sopha, Lenka Smid, Tomas Rousar, Jan M. Macak

**Affiliations:** † Department of Biological and Biochemical Sciences, Faculty of Chemical Technology, University of Pardubice, Studentska 573, 532 10 Pardubice, Czech Republic; ‡ Center of Materials and Nanotechnologies, Faculty of Chemical Technology, University of Pardubice, Nam. Cs. Legii 565, 530 02 Pardubice, Czech Republic; § Central European Institute of Technology, Brno University of Technology, Purkyňova 123, 61200 Brno, Czech Republic; ∥ Department of General and Inorganic Chemistry, Faculty of Chemical Technology, University of Pardubice, Studentska 573, 532 10 Pardubice, Czech Republic

**Keywords:** TiO_2_ nanotube layers, Ti foils, Ti-6Al-4V alloy, atomic layer deposition, cell proliferation, MG-63 cells

## Abstract

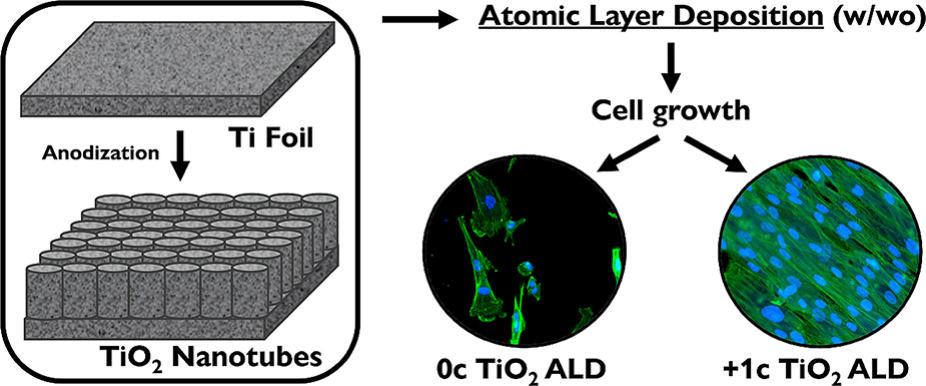

This work aims to investigate the chemical and/or structural modification of Ti and Ti-6Al-4V (TiAlV) alloy surfaces to possess even more favorable properties toward cell growth. These modifications were achieved by (i) growing TiO_2_ nanotube layers on these substrates by anodization, (ii) surface coating by ultrathin TiO_2_ atomic layer deposition (ALD), or (iii) by the combination of both. In particular, an ultrathin TiO_2_ coating, achieved by 1 cycle of TiO_2_ ALD, was intended to shade the impurities of F- and V-based species in tested materials while preserving the original structure and morphology. The cell growth on TiO_2_-coated and uncoated TiO_2_ nanotube layers, Ti foils, and TiAlV alloy foils were compared after incubation for up to 72 h. For evaluation of the biocompatibility of tested materials, cell lines of different tissue origin, including predominantly MG-63 osteoblastic cells, were used. For all tested nanomaterials, adding an ultrathin TiO_2_ coating improved the growth of MG-63 cells and other cell lines compared with the non-TiO_2_-coated counterparts. Here, the presented approach of ultrathin TiO_2_ coating could be used potentially for improving implants, especially in terms of shading problematic F- and V-based species in TiO_2_ nanotube layers.

## 1 Introduction

Metals have been used extensively in biomedical applications. Particularly, titanium (Ti) and its alloys have been widely used for orthopedics and dental implants mainly due to their exceptional corrosion and tribocorrosion resistance, excellent mechanical properties, and biocompatibility.^1,2^ The commercially pure Ti and the Ti-6Al-4V alloy are the most used in biomedical implants.^3,4^ For the classical pure Ti, the passive film on its surface is responsible for the recognized corrosion resistance and enhanced biocompatibility, which is predominantly composed of amorphous TiO_2_ and small amounts of suboxides, such as TiO and Ti_2_O_3_.^5,6^ On the other hand, the passive film on Ti-6Al-4V surfaces is composed of a compact inner TiO_2_ layer and a porous layer composed of TiO_2_, enriched with Al_2_O_3_, and V_2_O_5_ oxides in small amounts in the outermost passive film.^5,6^

Despite the wide success and usefulness of Ti implants, they may also suffer from degradation processes, such as wear and tribocorrosion, inducing the release of metallic particles and/or ions commonly associated with infections and allergies. As a matter of fact, Ti implants tend to be encapsulated by fibrous tissue, which may compromise the stability of the implant and cause implant loosening.^7−9^

In recent years, some experimental works have shown the benefit of surface modifications of implants by using nanostructured layers.^10−13^ The reason for this modification is to enhance bone cell proliferation, adhesion, differentiation, and mineralization around the implant, significantly improving the osseointegration. Surfaces of Ti and its alloys can be modified by the plasma electrolytic oxidation (PEO) process,^14,15^ or anodic oxidation,^16^ producing a stable TiO_2_ layer that highly facilitates osseointegration. TiO_2_ nanotube (TNT) layers represent one of the most attractive TiO_2_ materials, due to their high surface area, surface chemistry, and hydrophilicity, which all together allow the cells to adhere very well on their surface.^17−19^ Anodic oxidation allows tailoring of the features of the TNT layers such as tube diameter, thickness, and chemical composition. However, the Ti-6Al-4V alloy (and other alloys as well) contains other elements, in this case, Al (6 wt %) and V (4 wt %) that may affect the cell growth and cause cytotoxicity, leading possibly to unwanted events such as inflammatory responses, neurological, and non-neurological damages.^20−22^ These species are present in small amounts on the alloy, even when anodized having a nanotubular surface on the very top. In fact, species from electrolytes, such as F species, can also be left within nanotube walls and cause toxicity issues.^23^ The solution to this appears more and more to be the atomic layer deposition (ALD) technique, which is an effective technique to produce TiO_2_ coatings with precise control of the thickness and without virtually any change in the surface morphology. TiO_2_ ALD coating improves water resistance^24,25^ and mechanical properties.^26,27^ Such efforts lead to an increase in interfacial biocompatibility.^23^ Enhanced cell adhesion and proliferation can be explained by the increased surface roughness and hydrophilicity of the TiO_2_-coated samples, which allows the cells to adhere in greater numbers due to the higher cell-surface interactions.^28^ Low-temperature ALD is an important technique to functionalize and modify heat-sensitive biomaterials that could be thermally degraded.^29^ A recent study has demonstrated improved biocompatibility through the addition of a 50 nm-thick layer of TiO_2_ on polyetheretherketone.^30^ The material was tested on the mesenchymal tumor stem cell line ST-2, showing excellent osteoconductive properties. However, achieving such a thickness in ALD involves an extensive process with multiple cycles.^31−34^ Nevertheless, ultrathin TiO_2_ ALD coatings can effectively shade potentially poisonous elements, such as F and V, which can be detrimental to biocompatibility properties, decreasing the ALD process time. In our previous work, it was shown that ALD-modified TNT layers with 5c and 150c of TiO_2_ can significantly improve the cell growth and the biocompatibility of WI-38 fibroblasts by 50%, SH-SY5Y neuroblasts by 30%, and MG-63 osteoblasts by 30% compared with the uncoated counterparts.^23,35^ The comparison to Ti foils with a native and thermal oxide layer was also carried out.^23^

In the present study, a more in-depth study is intended, exploiting the cellular response to crystalline nanotubular oxides on Ti foils and also to amorphous oxides on biomedical Ti alloy (Ti-6Al-4V), which goes significantly beyond our previous work.^23^ This research aims to improve the cell growth on surfaces of metallic Ti and Ti-6Al-4V foils in their as-delivered or anodized states (i.e., with TNT layers on their surface) by adding ultrathin (1c, which correspond nominally to a thickness of ≈0.05 nm) TiO_2_ coating achieved by ALD process, which effectively reduces the presence of impurities, specifically F and V species, in all the nanomaterials tested while preserving their original structure and morphology. For that, Ti foils, Ti-6Al-4V foils, and TNT layers grown on Ti foils with and without TiO_2_ coatings were used to culture different human cell lines such as MG-63, WI-38, A549, U-87 MG, and MRC-5. In addition, TNT layers on Ti foils were investigated in the amorphous and crystalline (anatase) states, provided that these crystalline layers have not been yet exploited with ALD coatings. The entire set was characterized in terms of morphology, crystallinity, surface roughness, and wettability by SEM, XRD, EDS, XPS, AFM, profilometry, and static water contact angle (WCA), respectively. Then, the cell growth on all tested materials was characterized by fluorescence microscopy.

## 2 Experimental Details

### 2.1 Synthesis and Characterization of Materials

Prior to all experiments, the Ti (Merck, 0.127 mm thick, 99.7% purity) and Ti-6Al-4V alloy (Goodfellow, 0.1 mm thick, grade 5) foils were cut into square pieces (1.5 × 1.5 cm^2^), then degreased by sonication in acetone and isopropanol in an ultrasonic bath for 1 min, respectively, and then dried in a N_2_ jet. TNT layers were prepared via electrochemical anodization on the Ti foils. The TNT layers were grown at room temperature, and a Pt foil was used as a counter electrode. All anodizations were carried out with a high-voltage potentiostat (HEIDEN, EA-PSI 9200–15, Germany) attached to a digital multimeter (Keithley 2100, USA) in a glycerol-based electrolyte containing 50 vol % water and 0.27 M NH_4_F at 4 V for 3 h to obtain TNT layers of a distinct diameter. After the anodization, the TNT layers were sonicated in isopropyl alcohol and dried in air. The as-received TNT layers were amorphous and were further noted as AM-TNT layers.

To obtain a crystalline thermal oxide layer of TiO_2_ on Ti foils (further noted as CR-Ti) and crystalline TNT layers (further noted as CR-TNT layers), part of the Ti foils and AM-TNT layers were annealed at 400 °C for 1 h in a static atmosphere of air in a laboratory muffle oven, with a sweep rate of 2.1 °C min^–1^. For Ti-6Al-4V foils, the samples develop a passive film due to environmental conditions, further referred to as AM-TiAlV.

A part of the Ti and Ti-6Al-4V foils and TNT layers were coated by an ultrathin TiO_2_ coating using atomic layer deposition (ALD, TFS200, Beneq). The process was carried out at 300 °C using TiCl_4_ (electronic grade 99.9998%, STREM) as the Ti precursor and Millipore deionized water (18 MΩ) as the oxygen source. High-purity N_2_ (99.9999%) was the carrier and purging gas at a flow rate of 400 standard cubic centimeters per minute (sccm). Under these deposition conditions, one ALD growth cycle was defined by the following sequence: TiCl_4_ pulse (500 ms)–N_2_ purge (3 s)–H_2_O pulse (500 ms)–N_2_ purge (4 s). The corresponding layers are later denoted as +1c TiO_2_. The nominal thickness of 1c TiO_2_ is 0.055 nm according to our previous studies and reference measurements described there.^23^ The executive overview of all Ti and Ti-6Al-4V foils and TNT layers investigated in this study is given in Table 1.

**Table 1:** Overview of Ti and Ti-6Al-4V Foils and TNT Layers Investigated in This Study

**substrate**	**heat treatment**	**oxide structure**	**surface modification**	**abbreviation used in the text**
Ti foil	annealed	crystalline		CR-Ti
			1 ALD TiO_2_ cycle	CR-Ti+1c TiO_2_
	non-annealed	amorphous	TNT	AM-TNT
			TNT + 1 ALD TiO_2_ cycle	AM-TNT+1c TiO_2_
	annealed	crystalline	TNT	CR-TNT
			TNT + 1 ALD TiO_2_ cycle	CR-TNT+1c TiO_2_
Ti-6Al-4V foil	non-annealed	amorphous		AM-TiAlV
			1 ALD TiO_2_ cycle	AM-TiAlV+1c TiO_2_

The surface morphology of CR-Ti and AM-TiAlV foils and TNT layers was characterized by field-emission scanning electron microscopy (FE-SEM, JEOL JSM 7500F). The dimensions of TNT layers were evaluated by statistical analyses of SEM images using proprietary Nanomeasure software. The quantitative EDX measurements were performed on an electron microscope (LYRA3, Tescan) equipped with EDX analyzer AZtec X-Max 20 (Oxford Instruments) at an acceleration voltage of 20 kV.

The surface chemical composition of all CR-Ti, AM-TiAlV and TNT layers was evaluated by X-ray photoelectron spectroscopy (XPS, ESCA 2SR, Scienta Omicron) using a monochromatic Al Kα (1486.7 eV) X-ray source. The X-ray source was operated at 250 W. The binding energy scale was referenced to adventitious carbon (284.8 eV). No charging neutralizer was used during the measurements. The spectra were fitted using a Shirley-type background by CasaXPS software. The quantitative analysis was performed using the elemental sensitivity factors provided by the manufacturer.

The wettability of all the materials was evaluated by measuring the static water contact angle (WCA) using a Surface Energy Evaluation System device (see System E, Advex Instruments) with proprietary image analysis software. The measurements were carried out at room temperature using 3 μL droplets contacting the surfaces, and 5 s was allowed to stabilize. The contact angles of the water droplets were fitted by using the tangent line analysis method. Measurements were performed at 5 different positions on each material. All results were expressed as the mean ± standard deviation (SD).

The surface topography was analyzed by a digital holographic microscope (DHM, DHMR1000, Lyncee Tec, Switzerland) operating at 785 nm in reflection configuration on the scale of 170 μm and by atomic force microscopy (AFM, Solver Pro-M, NT-MDT, Russia) on an area of 5 × 5 μm^2^. The details of measurement and the statistical approach of roughness analysis labeled by RMS (root-mean-square) are in our previous articles.^23,36^

### 2.2 Cell Culture

Human osteoblast-like cells MG-63 (ATCC No.CRL-1427; doubling time, DT = 31 h), human lung fibroblast cells WI-38 (ATCC No. CCL-75; DT = 60 h), and diploid cell culture line composed of fibroblasts MRC-5 (ATCC No. CCL-171; DT = 45 h) were cultured in minimum essential medium (Merck) with 10% (v/v) fetal bovine serum (Gibco), 2 mmol·L^–1^ glutamine, 1% nonessential amino acids solution, and 50 μg·mL^–1^ penicillin/streptomycin solution (Gibco). Human lung carcinoma epithelial cells A549 (ATCC No. CCL-185; DT = 25 h) were cultured in minimum essential medium with 10% (v/v) FBS, 2 mmol·L^–1^ glutamine, 1 mmol·L^–1^ pyruvate, and 50 μg·mL^–1^ penicillin/streptomycin and maintained at 37 °C in a sterile humidified atmosphere of 5% CO_2_. Glioblastoma cells U-87 MG (ATCC No. HTB-14; DT = 27 h) were cultured in Dulbecco’s modified Eagle medium (Merck) with 15% (v/v) fetal bovine serum (Gibco) and 50 μg·mL^–1^ penicillin/streptomycin solution (Gibco) followed by incubation in an atmosphere of 5% CO_2_ at 37 °C. Cells were proven to be mycoplasma-free, and STR analysis confirmed the origin of all cell lines.

### 2.3 Cell Growth on Materials

Before any further use, the square-shaped substrates were cut into round shapes with a diameter of approximately 5 mm (using sharp scissors) to fit into the wells used for the cell growth. All of the tested materials were sterilized in 70% ethanol for 30 min, washed with deionized water, and dried. The foils were then placed on eight-well chamber slides. Briefly, 200 μL of a suspension of MG-63, WI-38, A549, U-87 MG, and MRC-5 cells with a density of 3 × 10^3^, 1 × 10^4^, 7 × 10^3^, 4 × 10^3^, and 8 × 10^3^ cells/cm^2^, respectively. The cells were added to each well of a chamber slide and were seeded and cultured for 24, 48, and 72 h. The cell densities were used to maintain the optimal cultivation conditions for up to 72 h. To visualize actin filaments and cell nuclei, phalloidin-FITC and Hoechst 33258 dyes were used, respectively. After being seeded for 24, 48, and 72 h, cultured cells were fixed with 3.7% formaldehyde (5 min; 37 °C; dark) and permeabilized with 0.1% Triton X-100 (15 min; 37 °C; dark). Then, 100 μL of phalloidin-FITC (1 μmol·L^–1^) was added and the samples were incubated for 40 min at 37 °C. Ten minutes before the end of phalloidin-FITC loading, 10 μL of Hoechst 33258 solution was added to cells. The final concentration of Hoechst 33258 in a well was 2 μg·mL^–1^. Then, the cells were washed twice with phosphate-buffered saline (37 °C). Actin filaments (FITC filter, 480/30 nm) and cell nuclei (DAPI filter, 375/28 nm) were observed with an Eclipse 80i fluorescence microscope (Nikon, Japan). The number of cells grown on the surface was counted from at least 35 fields of view using an NIS-Elements AR (Nikon, Japan). All experiments were repeated two times independently at least. The number of cell nuclei was related to 1 mm^2^ and expressed as mean ± standard error of the mean (SEM) taken from fluorescence images. Quantitative analysis of the elongation of all cell lines on tested samples was provided using an NIS-Elements AR (Nikon, Japan).

### 2.4 Statistics

All experiments were repeated at least three times independently. The number of fields was *n* = 35. The results are expressed as the mean ± SD. Statistical significance was analyzed after normality testing using a one-way ANOVA test followed by a Bonferroni post-test (OriginPro 9.0.0, USA) to compare results to each other at significance level *p* = 0.05.

## 3 Results and Discussion

### 3.1 Surface, Structure, and Composition Characteristics

**Figure 1:**
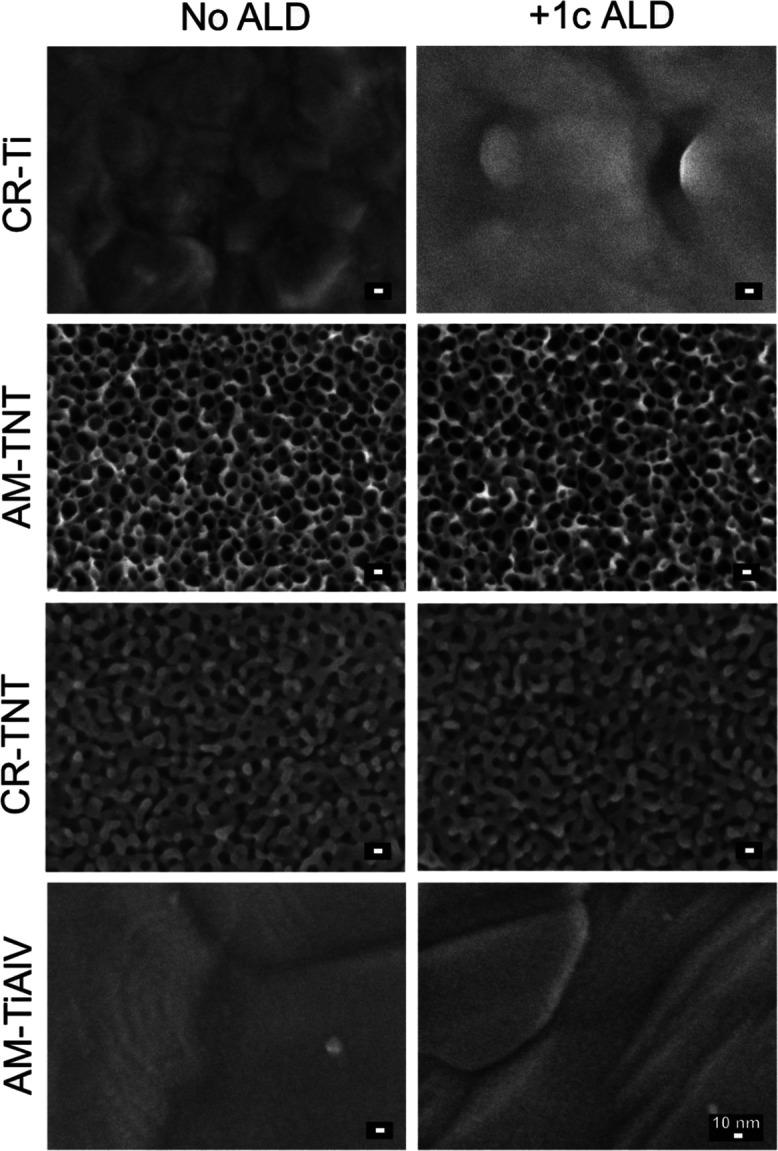
SEM top-view images of CR-Ti and AM-TiAlV foils and TNT layers (CR = crystalline; AM = amorphous). The left column shows uncoated surfaces, and the right column surfaces with ultrathin 1c TiO_2_ coatings by ALD.

Top-view SEM images of all of the foils and TNT layers are depicted in Figure 1. The left column shows uncoated surfaces, while the right column shows all surfaces after the ALD process of 1c TiO_2_. As one can see by comparison of the columns, the coating of neither foils nor TNT layers with 1c TiO_2_ did not result in any morphological changes. This is due to the fact, that the coating is ultrathin (nominal thickness of 0.055 nm).^23^ Upon a close look at all CR-Ti and AM-TiAlV foils and TNT layers, one can state that CR-Ti foil exhibits a typical rough surface of a metallic rolled foil, while the AM-TiAlV alloy reveals a surface with a microstructure of equiaxed grains. As a result of the anodization, self-organized TNT layers were obtained on the Ti foils. Upon detailed measurements, inner diameters of ∼15 nm for AM-TNT and ∼11 nm for CR-TNT layers were revealed. The layer thicknesses of the corresponding AM-TNT and CR-TNT layers, determined from SEM cross-sectional images, were ∼280 and ∼175 nm, respectively (Figure S1a,b). After annealing, the surface and the thickness of the CR-TNT layers may change due to sintering and shrinking, resulting in a smaller inner diameter and thinner TNT layers after annealing.

The surface chemical composition in atomic % was evaluated by XPS (survey spectra, Figure S2), and the results are depicted in Table 2. Some differences in the surface atomic composition of uncoated and 1c TiO_2_-coated CR-Ti and AM-TiAlV foils and TNT layers were observed. For all 1c TiO_2_-coated foils and TNT layers, the amount of F and Al (that stem from the anodization electrolytes used and the composition of the alloy) decreased, while the Ti and O increased compared to the uncoated foils and TNT layers.^37^ This is due to the shading created by the addition of a homogeneous layer of TiO_2_ on the entire surface, achieved by a robust ALD protocol that provides enough precursors and time to grow a uniform layer.^23^ Other elements like C (adventitious carbon) and N (stemming from the anodization electrolyte and surface contaminants of the foils) did not display significant changes after 1c TiO_2_-coated. It was not possible to detect the V on the surface of the foils; however, it was found in the bulk by EDX, as shown in Table S1. Even though the XPS is a sensitive surface technique with a depth interaction only of a few nanometers (∼5–10 nm), the addition of such an ultrathin compact TiO_2_ layer (as created by 1 ALD cycle) will not entirely prevent the detection of other species underneath the coating. The slight increase of O and Ti in 1c TiO_2_-coated TNT layers is evidence of the high purity of 1c TiO_2_ (in other words, more stoichiometric TiO_2_ than the nanotubular TiO_2_).

**Table 2:** XPS Data Showing the Surface Chemical Composition in atomic % of Uncoated and 1c TiO_2_-Coated CR-Ti Foil, AM-TNT and CR-TNT Layers, and AM-TiAlV

**chemical composition (atomic %)**								
**substrate**	C	O	F	Al	Ti	V	N	other
CR-Ti	31.19	42.86	3.19		18.67		3.61	0.48
CR-Ti+1c TiO_2_	32.09	44.57			19.66		3.11	0.57
AM-TNT	29.91	47.29	4.10		17.15		1.55	
AM-TNT+1c TiO_2_	29.98	49.95	0.92		17.75		1.40	
CR-TNT	15.76	59.24	1.07		23.30		0.63	
CR-TNT+1c TiO_2_	19.76	56.92	0.83		21.63		0.77	0.09
AM-TiAlV	43.81	35.31		3.33	11.50		3.25	2.80
AM-TiAlV+1c TiO_2_	43.20	37.22		2.90	11.76		3.18	1.74

### 3.2 Wettability Measurements

Surface wettability significantly influences biocompatibility, such as cellular adhesion, morphology, metabolic activity, and proliferation.^38,39^Table 3 and Figure S3 show the WCA values and contact angles of water droplets for all materials before and after 1c TiO_2_, respectively. The deposition of 1c TiO_2_ led to an improvement of the hydrophilic nature of the surface, as indicated by the lower WCA values measured for all materials evaluated, except the CR-TNT samples. The lowering of the contact angle of TiO_2_ surfaces after the ALD process of TiO_2_ was also observed in the literature.^40^ The reason for the CR-TNT exception against the observed trend must be examined in future work.

**Table 3:** Contact Angles of Water Droplets on Uncoated and 1c TiO_2_-Coated CR-Ti, AM-TNT, CR-TNT, and AM-TiAlV

**substrate**	**No ALD**	**+ 1c TiO**_**2**_
CR-Ti	70.7 ± 1.7°	65.5 ± 2.4°
AM-TNT	110.1 ± 2.4°	62.9 ± 10.7°
CR-TNT	46.0 ± 2.8°	63.5 ± 1.1°
AM-TiAlV	97.9 ± 1.5°	88.6 ± 1.9°

### 3.3 Surface Topography and Roughness

**Figure 2:**
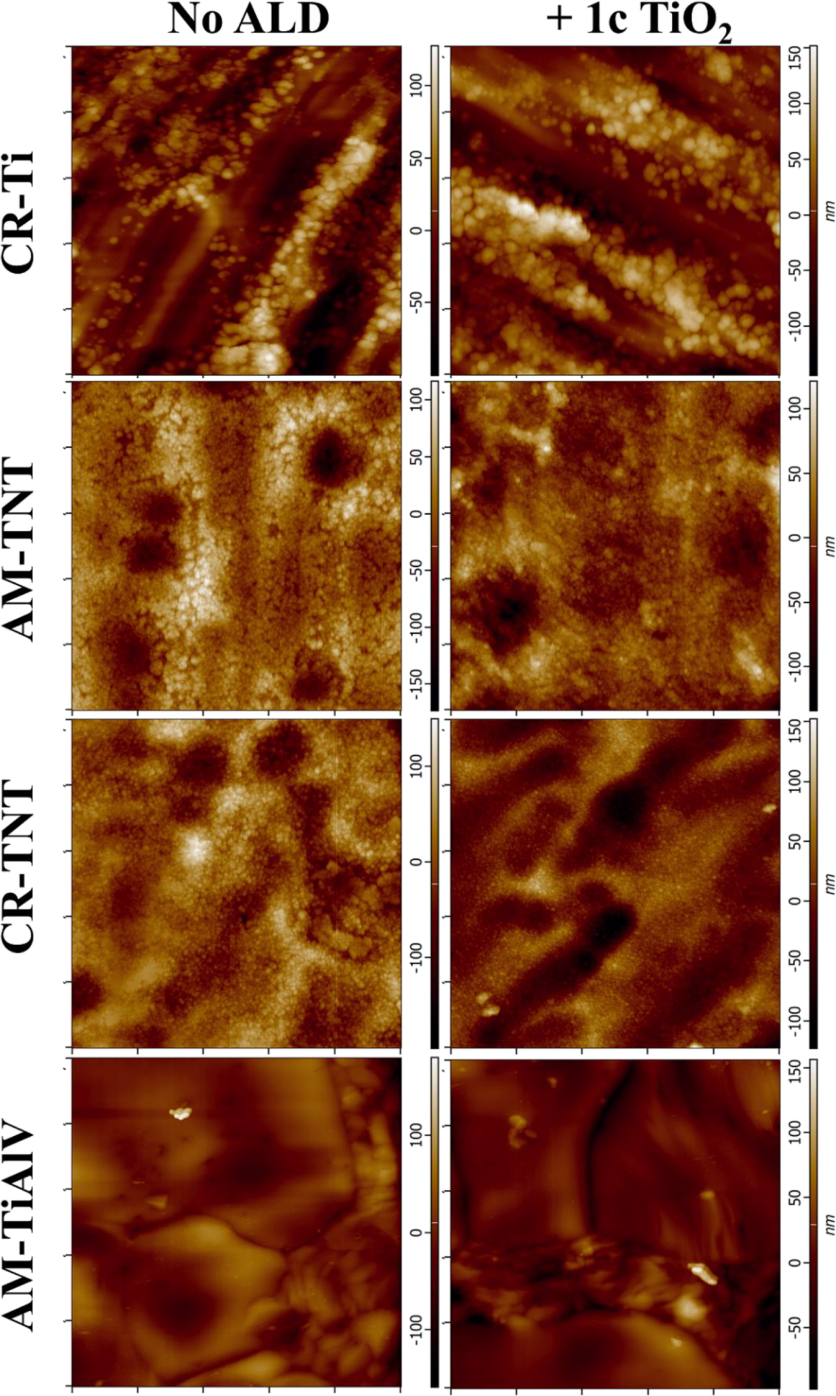
AFM topological images of the studied materials. The left column shows uncoated surfaces, and the right column surfaces with 1c TiO_2_-coated (CR = crystalline; AM = amorphous). The scan area is 5 × 5 μm^2^ for all images.

All nanomaterials used in this work were subjected to the measurement of surface topography by means of AFM (scale 5 μm) and DHM (scale 170 μm). The AFM topographical images are shown in Figure 2. The AFM topological images of the studied materials in the 3D visualization are shown in Figure S4. While no metallic grains are visible on surfaces that were thermally treated (i.e., thermal oxide grew on them) or anodized (TNT layer grew on them), one can clearly see that the Ti alloy (AM-TiAlV) displays the typical metallic grains. The TiO_2_ ALD deposition did not affect the surface morphology of any of the samples investigated as visualized by AFM images (right column).

**Figure 3:**
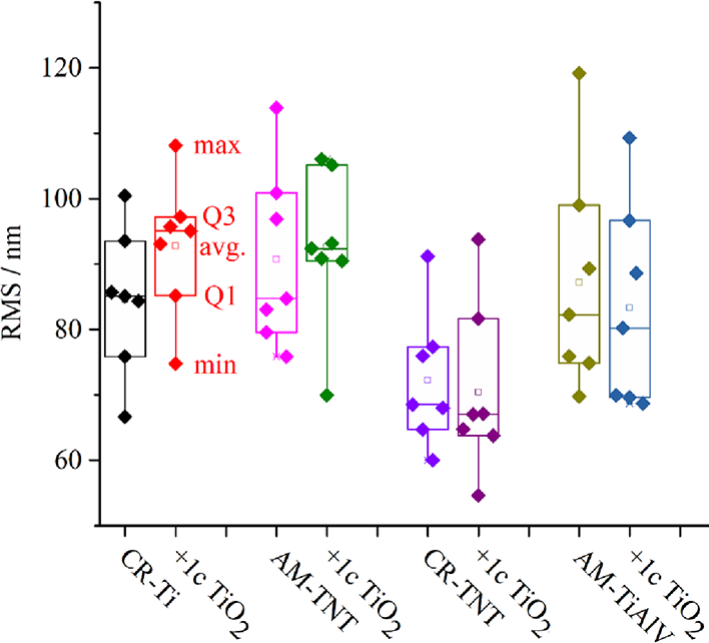
Roughness values (root-mean-square, RMS) obtained by DHM in the form of the box plot describing mean (open square); first and third quantile (box); and min/max values (whisker) of uncoated and 1c TiO_2_-coated materials. The dotted rectangle reflects the experimental RMS value for each line.

In Figure S5, one can see the illustration of the surface topography at a scale >170 μm, obtained by non-contact DHM profilometry. Figure 3 shows the resulting root-mean-square (RMS) values of the surface roughness, statistically represented as a box plot. The average values of the RMS depicted in Table 4 were in a narrow range of 70–92 nm. The ALD coating (1c of TiO_2_, nominal thickness of ≈0.05 nm) did not change the roughness values (RMS) on the scales of >170 and 5 μm within the experimental error.

**Table 4:** Roughness (RMS) Values for the Studied Material Determined at the Large (DHM - 170 μm^a^) and Small (AFM −5 μm^b^) Scales

**substrate**	**RMS (nm)^a^**	**RMS (nm)^b^**
CR-Ti	86	38
CR-Ti + 1c TiO_2_	92	35
AM-TNT	91	36
AM-TNT + 1c TiO_2_	93	34
CR-TNT	72	30
CR-TNT + 1c TiO_2_	70	27
AM-TiAlV	88	35
AM-TiAlV + 1c TiO_2_	83	37

a DHM based analysis (170 μm) with standard error of mean = 4 nm.

b AFM-based analysis (5 × 5 μm^2^) with standard error of mean = 3 nm.

### 3.4 Cell proliferation – Ti Foils and TNT Layers

In this section, the evaluation of growth, i.e., adhesion and proliferation, of MG-63 cells during incubation up to 72 h was carried out on 1c TiO_2_-coated CR-Ti foils and both CR- and AM-TNT layers (TNTs ∼ 15 nm diameter). The MG-63 cell line was selected for the initial cellular experiment because MG-63 cells have been frequently used to evaluate the cell growth on surfaces modified using the ALD technique based on their bone origin.^23,41,42^ In principle, the present study follows the recent report showing the beneficial effect of 5c TiO_2_-coated nanomaterials on cell growth and proliferation compared to 150c TiO_2_.^23^ In addition, the crystalline nanotube layers were included in this study.

**Figure 4:**
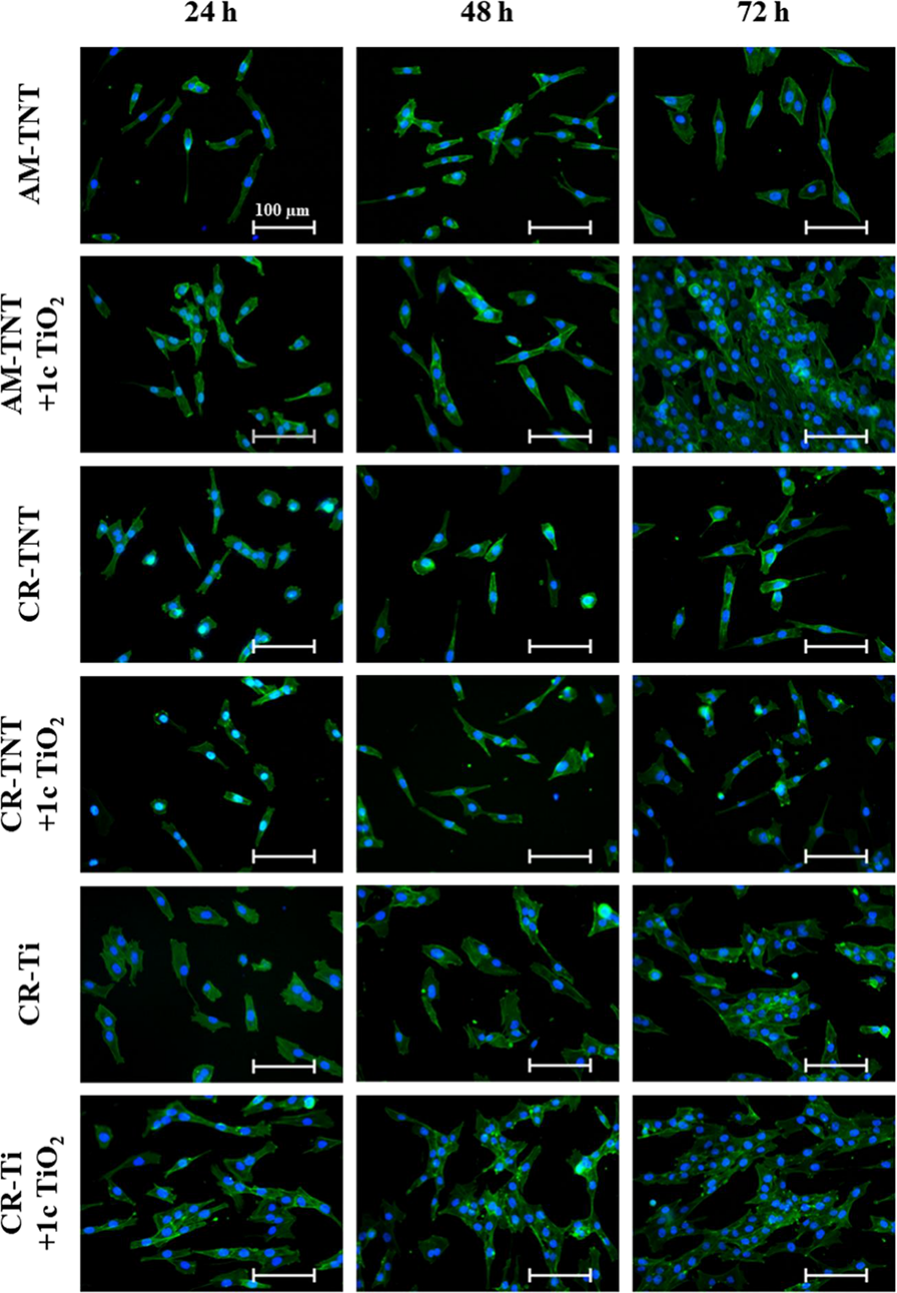
Photomicrographs of MG-63 cells on uncoated or 1c TiO_2_-coated CR-Ti foils, CR-TNT, and AM-TNT layers grown for 24–72 h (CR = crystalline; AM = amorphous; 1c TiO_2_ = 1c TiO_2_ ALD coating). The actin filaments were stained with the phalloidin-FITC probe (green), and the cell′s nuclei were stained with the Hoechst 33258 probe (blue).

MG-63 cells were cultured on CR-Ti foils and AM/CR-TNT layers both uncoated or coated with 1c TiO_2_ ALD. Fluorescence staining of cell nuclei and actin filaments was used to identify the functional morphology of MG-63 cells depicted in Figure 4 according to several reports using the same approach.^33,43,44^ MG-63 cells incubated on AM/CR-TNT layers and CR-Ti foils coated with 1c TiO_2_ had elongated cell shapes compared to those on uncoated ones, especially after 48 and 72 h (Figure 4). To evaluate the cell interaction with 1c TiO_2_-coated foils and TNT layers, elongation of the cells was considered. The occurrence of elongated MG-63 cells reflects the proper adhesion of cells to the surface.^45^ Assessment of elongation MG-63 cells on uncoated or 1c TiO_2_-coated CR-Ti foils, CR-TNT, and AM-TNT layers is provided in the Supporting Information file (Figure S6). An increased elongation of MG-63 cells by approximately 20% was observed on all tested foils and TNT layers coated with 1c TiO_2_ ALD compared to that of uncoated ones. The elongated morphology of cells on ALD-coated samples was found also in previous studies.^46,47^ For instance, the elongated morphology of MC3T3-E1 osteoblasts on titanium sheets coated with hydroxyapatite by ALD was observed after 48 h, in contrast to the circular morphology exhibited when the cells were grown on cover glass.^46^ Other authors show that the human fetal mesenchymal stem cells grown on Ag nanoparticles and Ag/TiO_2_ nanostructures modified by ALD were elongated, bipolar, spindle-shaped, and interconnected by filopodia after 24 h.^47^

**Figure 5:**
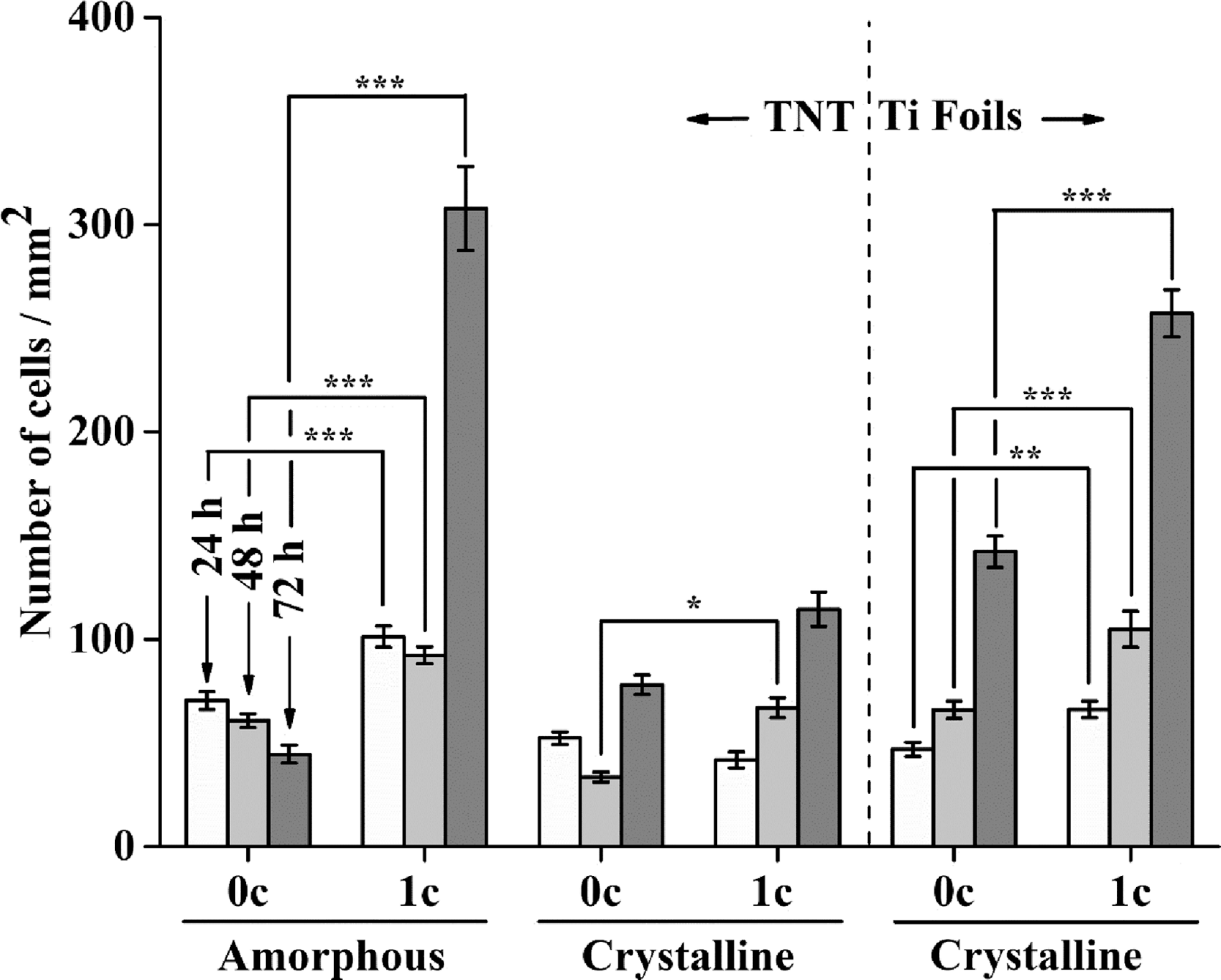
Density of MG-63 cells on crystalline thermal oxide layer on Ti foil and crystalline/amorphous TNT layers uncoated or 1c TiO_2_-coated grown for 24–72 h (0c = without TiO_2_ ALD coating; 1c TiO_2_ = 1c TiO_2_ ALD coating). Data originated from two independent experiments presented as mean ± SEM (*, *p* < 0.05; **, *p* < 0.01; ***, *p* < 0.001).

Those images were subjected to image analysis. Counting the cell nuclei in individual fields of view and relating them to the mm^2^ area gave a quantitative view of cell density in tested samples. This approach for counting cellular nuclei has been commonly used in several reports to assess the cell density and proliferation.^44,48,49^ An increase in the number of cells incubated on materials coated with 1c TiO_2_ in comparison to uncoated counterparts was observed at all time intervals, as shown in Figure 5. The most obvious increases in the cell density were found in AM-TNT layers and CR-Ti foils after 72 h, where the enhancement of cell density was approximately 7-times higher in comparison to uncoated layers or foils. Because the doubling time of MG-63 cells was 31 h, we can conclude that we characterized not only the cell adhesion but also the cell proliferation on the tested materials.

The surface chemistry together with the surface topography can affect surface wetting properties (hydrophilicity/hydrophobicity) that can further affect the cell proliferation on the surface.^40,50^ In terms of surface chemistry, the beneficial effect of 1c TiO_2_ ALD coating can be likely attributed to the shading of elements, such as fluorine, leading to considerable biocompatibility improvement.^23^

The WCA measurements provided in Table 3 show an enhanced surface wettability (higher hydrophilicity) after 1c TiO_2_ ALD for almost all modified surfaces, evidencing the beneficial effect on the cell proliferation activity in tested materials demonstrated in Figure 5. Herein, the influence of 1c TiO_2_ ALD coating is very difficult to compare with other reports.^33,34^ Other authors describe the direct effect of the TiO_2_ material coverage using the ALD technique (200–2500 cycles of TiO_2_) on cell proliferation.^33,34^ However, in contrast to the present study, those reports show cell proliferation in materials that yielded significantly thicker coatings, making a comparison not possible. Another important factor that may influence cell proliferation is the WCA. For instance, the impact of the WCA was observed for different kinds of surfaces, and cell proliferation was improved with WCA about 60°.^40,50−52^ However, the WCA is one of several factors that influence cell growth. Thus, care needs to be taken when judging the influence of the WCA on the surfaces presented in this work.

### 3.5 Cell proliferation – Ti-6Al-4V Foils

**Figure 6:**
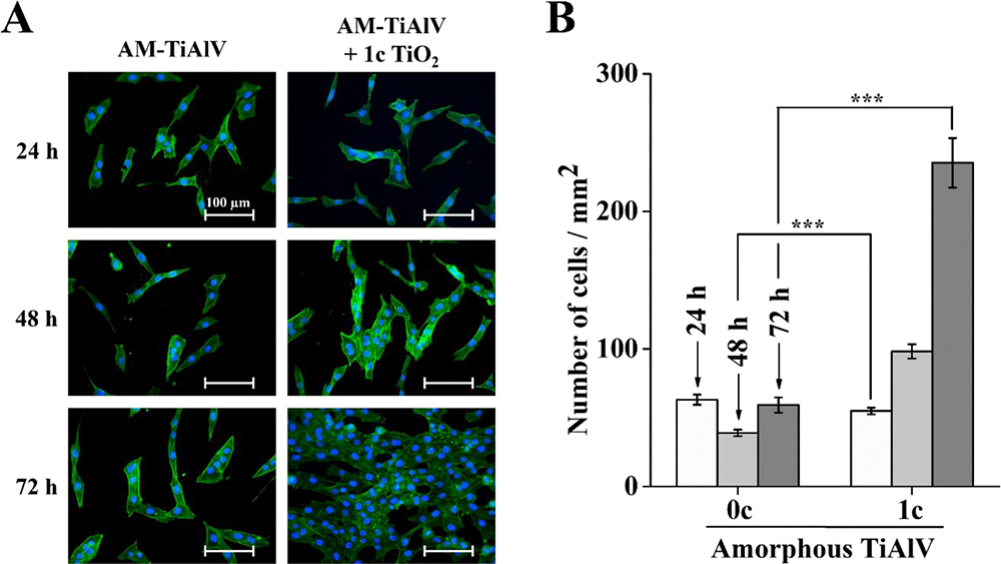
MG-63 cells on uncoated and 1c TiO_2_-coated AM-TiAlV foils grown for 24–72 h (AM = amorphous; 0c = without TiO_2_ ALD coating; 1c TiO_2_ = 1c TiO_2_ ALD coating). (A) Photomicrographs of MG-63 cells. The actin filaments were stained with the Phalloidin-FITC probe (green), and the cells’ nuclei were stained with the Hoechst 33258 probe (blue); (B) number of cells’ nuclei corresponding to cell count was quantified and presented in the graph. Data originating from two independent experiments are presented as mean ± SEM (***, *p* < 0.001).

The beneficial cellular effect of 1c TiO_2_ ALD coating in AM-TiAlV foils on the cell proliferation of MG-63 cells was evaluated. The relevance of the use of the MG-63 cell line was based on their frequent use in Ti-6Al-4V alloys.^53−56^Figure 6A shows the MG-63 cell growth on AM-TiAlV. Fluorescence staining of nuclei (blue) and actin filaments (green) was used to identify the functional morphology of the MG-63 cells. On AM-TiAlV + 1c TiO_2_, an elongated structure of the cells was observed compared to those cells cultured on the uncoated foils. The evaluation of the elongation of MG-63 cells on uncoated and 1c TiO_2_-coated AM-TiAlV foils is provided in the Supporting Information file (Figure S7). The occurrence of elongated MG-63 cells grown on AM-TiAlV coated with 1c TiO_2_ ALD in comparison with uncoated AM-TiAlV by approximately 15%, after 24 and 48 h was observed. The analysis of cell elongation after 72 h of incubation was not performed due to nearly 100% cell confluence. This finding became even more obvious over increasing time. The results from the optical analysis were used to quantify the number of cells present on a surface. Figure 6B shows 2.5-fold and 4-fold increases in the number of cells cultured on AM-TiAlV + 1c TiO_2_ compared to uncoated foils, after 48 and 72 h, respectively.

**Figure 7:**
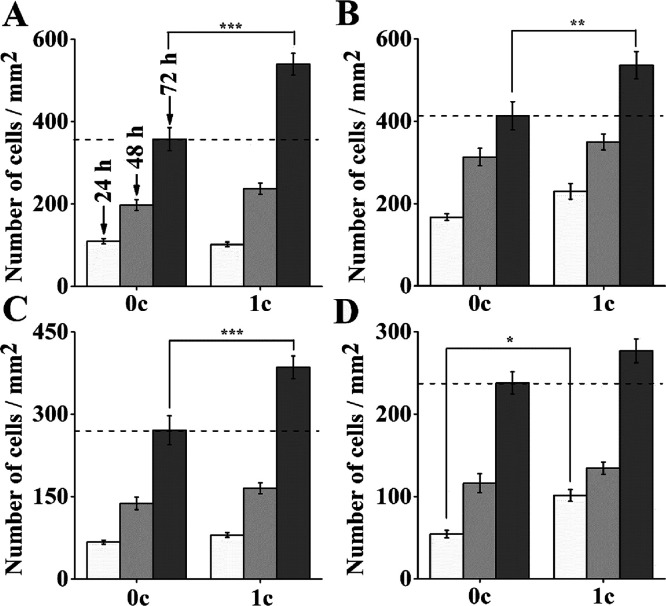
Density of various cell lines on uncoated and 1c TiO_2_-coated AM-TiAlV grown for 24–72 h (0c = without TiO_2_ ALD coating; 1c = 1c TiO_2_ ALD coating). (A) WI-38, (B) A549, (C) MRC-5, and (D) U-87 MG; data originated from two independent experiments are presented as mean ± SEM (*p < 0.05; **p < 0.01; ***p < 0.001).

In the last part of the present study, the cellular effect of 1c TiO_2_ ALD-coated AM-TiAlV in other cell lines was evaluated. Thus, four cell lines of different origin, shape, and size were used. Fibroblasts (WI-38, MRC-5), A549 pulmonary cells, and U-87 MG glial cells were used. The results revealed a significant enhancement of the cell growth on 1c TiO_2_ ALD-coated AM-TiAlV surfaces in all tested cell lines. According to outcomes presented in Figure 7, the most obvious increase in the number of cells incubated on uncoated or 1c TiO_2_ coated AM-TiAlV was observed after 72 h of incubation, i.e., 150% in WI-38, 130% in A549, 143% in MRC-5 and 117% in U-87 MG (comparing the cell growth on uncoated materials = 100%). A beneficial effect of TiO_2_ ALD coating was found in a recent study, in which TiAlV disks, which were chemically etched, covered with 2000c TiO_2_ by ALD, and polished, improved biocompatible, and allowed osteogenic differentiation of human mesenchymal stromal cells after 21 days.^57^ In our study, an increase in cell length was observed in all tested cell lines on AM-TiAlV coated with 1c TiO_2_ ALD in comparison with uncoated AM-TiAlV. Analysis of the elongation of A549, MRC-5, and U-87 MG cells on uncoated and 1c TiO_2_-coated AM-TiAlV is provided in the Supporting Information file (Figure S8). In the case of the MRC-5 cell line, the most beneficial effect of 1c TiO_2_-coated materials on cell elongation was observed. Analysis of the elongation WI-38 cell line after 72 h was not performed due to cell confluence of nearly 100%. The original fluorescence micrographs showing the beneficial effect of 1c TiO_2_ ALD coating on the cell growth of the different cell lines are provided in the Supporting Information file (Figures S9–S12). In conclusion, these results strongly confirmed our above-demonstrated data that the thinnest possible coating made by only 1c TiO_2_ ALD process changed the surface chemistry and wettability, resulting in enhanced cell proliferation.

## 4 Conclusions

In this work, surfaces of metallic Ti and Ti-6Al-4V foils were investigated as such or after anodization, yielding coverage by nanotubular layers. Then, they were coated by ultrathin coating with a nominal thickness of 0.055 nm, produced using 1 TiO_2_ ALD cycle. The surfaces were investigated in terms of the morphology, composition, and wettability. SEM, AFM, and profilometric results did not show any significant changes in the roughness and morphology of surfaces after 1c of TiO_2_ ALD coating in any of the substrates. The XPS analysis revealed that the chemical composition of all surfaces was modified after 1c TiO_2_ giving a decrease of occurrence of F and Al elements as a result. Then, the growth of MG-63 cells on all materials was estimated. In the case of Ti surfaces, AM-TNT + 1c TiO_2_ exhibited the highest MG-63 osteoblast cell density when compared to other Ti surfaces tested. Additionally, the cell proliferation was evaluated in other four cell lines for AM-TiAlV alloy surfaces. A significant increase in the number of cells growing on AM-TiAlV + 1c TiO_2_ compared with uncoated counterparts was found. In addition, we found the beneficial effect of 1c TiO_2_ ALD on cell elongation. The results on surface wettability showed that surface modification with a 1c TiO_2_ ALD coating decreases the surface hydrophilicity in almost all cases (except for CR-TNT) adjusting the contact angle between 60–80°. Thus, the occurrence of increased cell proliferation on surfaces coated with 1c TiO_2_ ALD coating can be caused by a combination of changing the chemistry of the surface and the wettability whereby the ultrathin TiO_2_ coating can diminish the negative cytotoxicity effect of F and V in the widely used biomedical Ti-6Al-4V alloy. The contributions of surface chemistry, surface structure, and wettability of the 1c TiO_2_ ALD-coated materials to cell proliferation seem to be equal. Overall, the results presented here bring new and very valuable information on the modification of biomedically relevant surfaces toward significantly improved biocompatibility and promoted cell growth.

## Supporting Information Available

SEM cross-sectional views of AM-TNT and CR-TNT layers, XPS survey spectra, EDX data, photographs of water droplets with contact angle values, topological 3D AFM images, topographical profiles obtained by profilometry of all layers uncoated and 1c TiO_2_-coated, analyses of cell elongations, photomicrographs of A549 and MRC-5 cells on uncoated and 1c TiO_2_-coated AM-TiAlV (PDF)
