# Supplementary material for: Ultrathin TiO2 Coatings via Atomic Layer Deposition Strongly Improve Cellular Interactions on Planar and Nanotubular Biomedical Ti Substrates
Source: ACS Appl Mater Interfaces. 2024 Jan 26;16(5):5627–36. doi: 10.1021/acsami.3c17074 (PMC10859894; doi:10.1021/acsami.3c17074)
Supplement: Supplementary file 1 — am3c17074_si_001.pdf [file am3c17074_si_001.pdf]

Supporting information

Ultrathin TiO_2 coatings via ALD strongly improve cellular interactions on planar and nanotubular biomedical Ti substrates

Jan Capek[1]†, Marcela Sepúlveda[2]†, Jana Bacova[1], Jhonatan Rodriguez-Pereira[2,3], Raul Zazpe[2,3], Veronika Cicmancova[2], Pavlina Nyvltova[1], Jiri Handl[1], Petr Knotek[4], Kaushik Baishya[3], Hanna Sopha[2,3], Lenka Smid[1], Tomas Rousar[1]* and Jan M. Macak[2,3]*

[1] Department of Biological and Biochemical Sciences, Faculty of Chemical Technology, University of Pardubice, Studentska 573, 532 10 Pardubice, Czech Republic

[2] Center of Materials and Nanotechnologies, Faculty of Chemical Technology, University of Pardubice, Nam. Cs. Legii 565, 530 02 Pardubice, Czech Republic

[3]Central European Institute of Technology, Brno University of Technology, Purkyňova 123, 61200 Brno, Czech Republic

[4] Department of General and Inorganic Chemistry, Faculty of Chemical Technology, University of Pardubice, Studentska 573, 532 10 Pardubice, Czech Republic

* Corresponding author (for the nanomaterial preparation and characterization):

Dr. Jan M. Macak, Center of Materials and Nanotechnologies, Faculty of Chemical Technology, University of Pardubice, Nam. Cs. Legii, 532 10 Pardubice, Czech Republic, ORCID: 0000-0001-7091-3022, Telephone: +420 466 037 401

E-mail: Jan.Macak@upce.cz

* Corresponding author (for the biological testing):

Assoc. Prof. Tomáš Roušar, Department of Biological and Biochemical Sciences, Faculty of Chemical Technology, University of Pardubice, Studentska 573, 532 10 Pardubice, Czech Republic. ORCID: 0000-0002-6893-821X, Telephone: +420 466 037 707

E-mail: Tomas.Rousar@upce.cz

† These authors contributed equally.

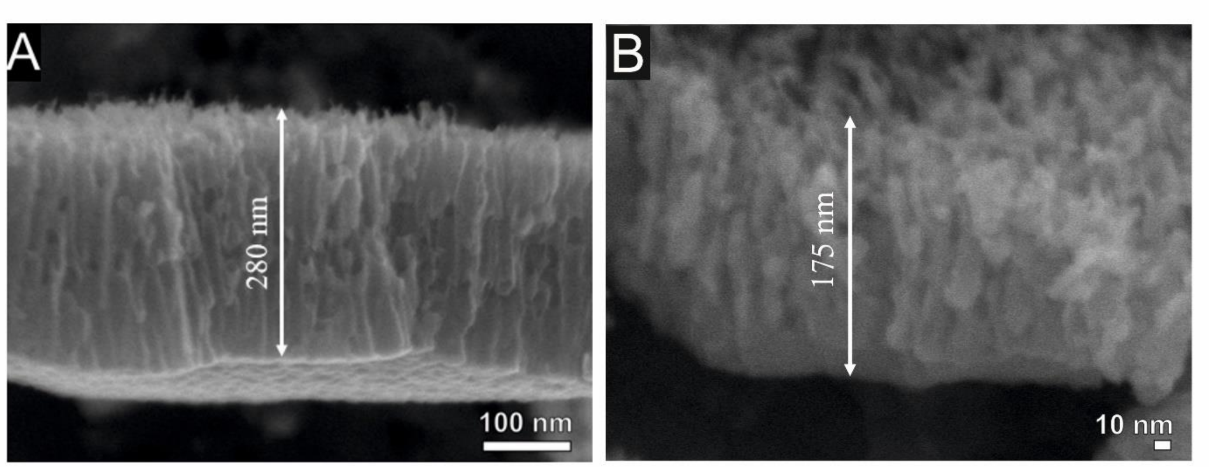

Figure S1. SEM cross-sectional views of A) AM-TNT and B) CR-TNT layers obtained on Ti foils.

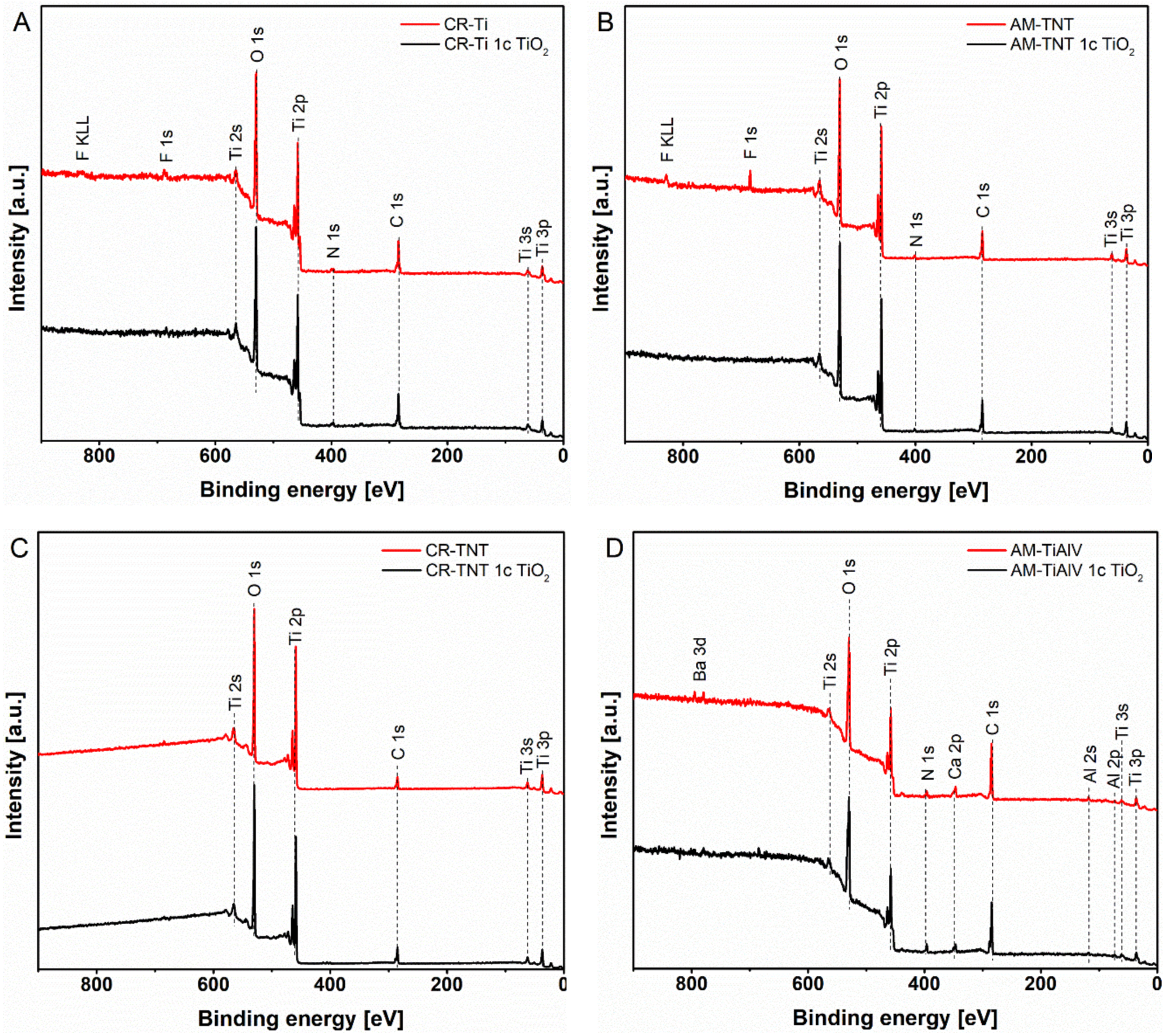

Figure S2. XPS survey spectra of all layers uncoated and 1c TiO_2-coated: A. CR-Ti, B. CR-TNT, C. AM-TNT, and D. AM-TiAlV foils.

Table S1. EDX data showing the chemical composition in atomic % of uncoated and ALD 1c TiO_2-coated CR-Ti and AM-TiAlV foils, TNT layers amorphous and crystalline states.

**Chemical composition (atom %)**						
**Substrate**	**C**	**O**	**F**	**Al**	**Ti**	**V**
**CR-Ti**	7.27	13.66	-	-	79.07	-
**CR-Ti + 1c TiO_2**	7.29	14.14	-	-	78.57	-
**AM-TNT**	4.16	46.48	5.24	-	43.91	-
**AM-TNT+ 1c TiO_2**	3.82	47.28	1.89	-	47.23	-
**CR-TNT**	0.87	54.04	1.05	-	44.03	-
**CR-TNT+ 1c TiO_2**	21.98	37.98	0.7	-	39.33	-
**AM-TiAlV**	1.85	-	-	9.90	85.01	3.24
**AM-TiAlV + 1c TiO_2**	2.31	-	-	9.86	85.03	3.12

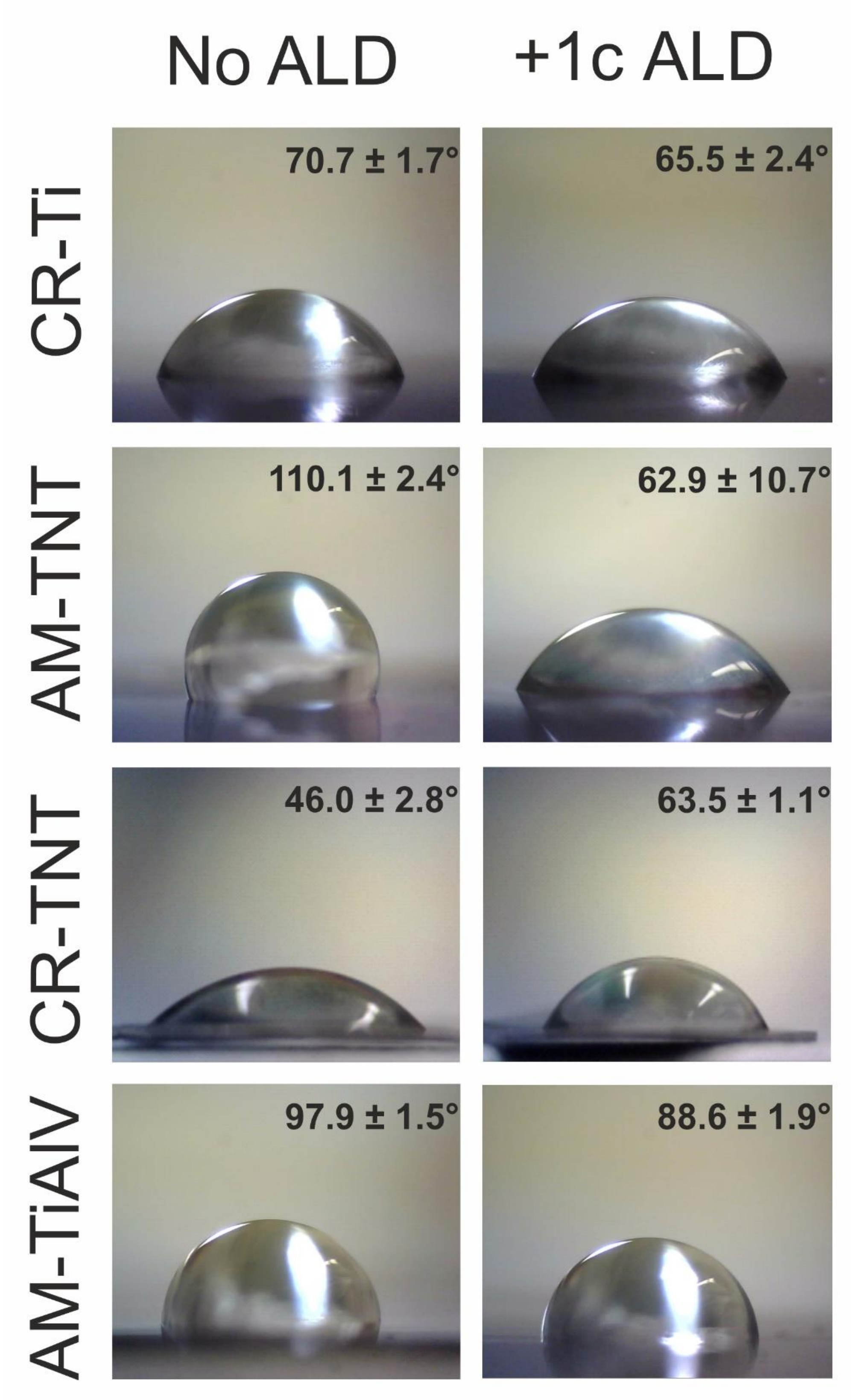

Figure S3. Contact angle values and water droplets on uncoated and ALD 1c TiO_2-coated CR-Ti and AM-TiAlV foils, and TNT layers in amorphous and crystalline states.

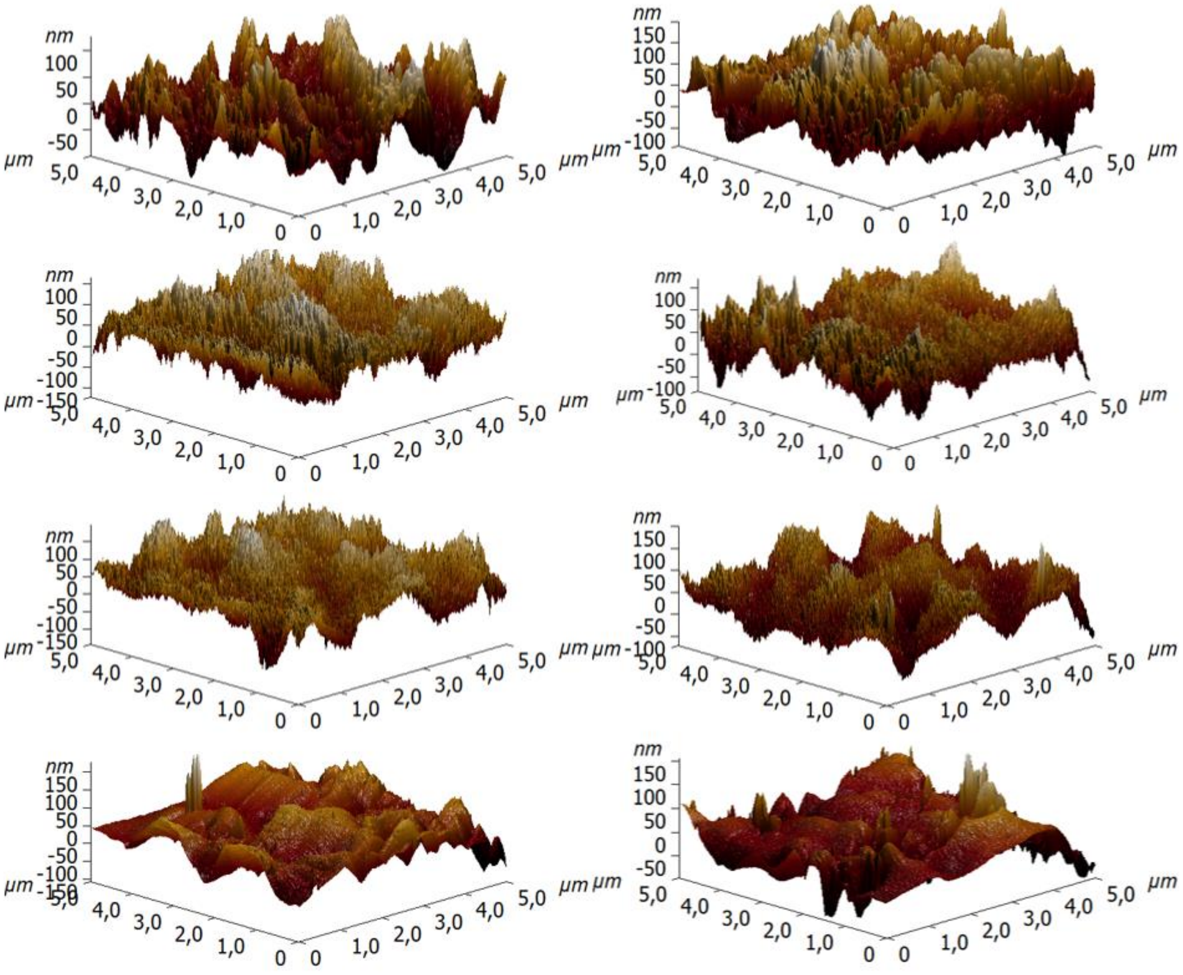

Figure S4. AFM topological images of the studied materials in 3D visualization. The left column shows uncoated surfaces and the right column surfaces with 1c TiO_2-coated (CR = crystalline; AM = amorphous). The images correspond to the Figure 2.

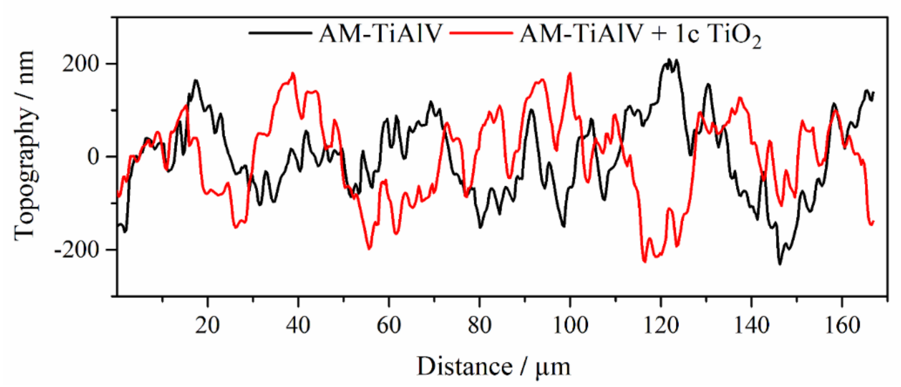

Figure S5. Illustration of topographical profiles obtained by profilometry on uncoated and 1c TiO_2-coated AM-TiAlV substrates.

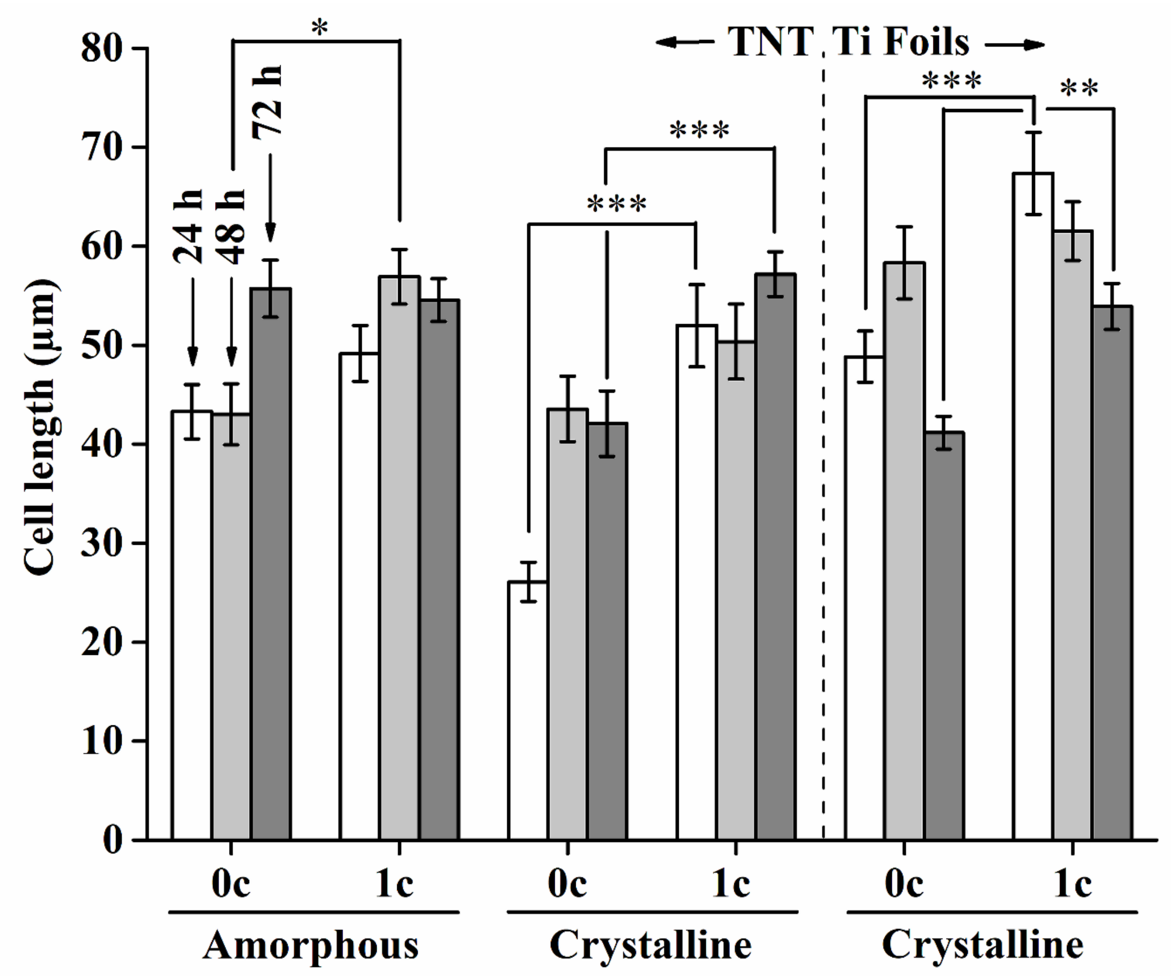

Figure S6. Analysis of elongation of MG-63 cells on uncoated or 1c TiO_2-coated CR-Ti foils, CR-TNT, and AM-TNT layers grown for 24-72 h (CR = crystalline; AM = amorphous; 1c TiO_2 = 1c TiO_2 ALD coating). The data are presented as mean ± SD (*, $p < 0.05$; **, $p < 0.01$; ***, $p < 0.001$).

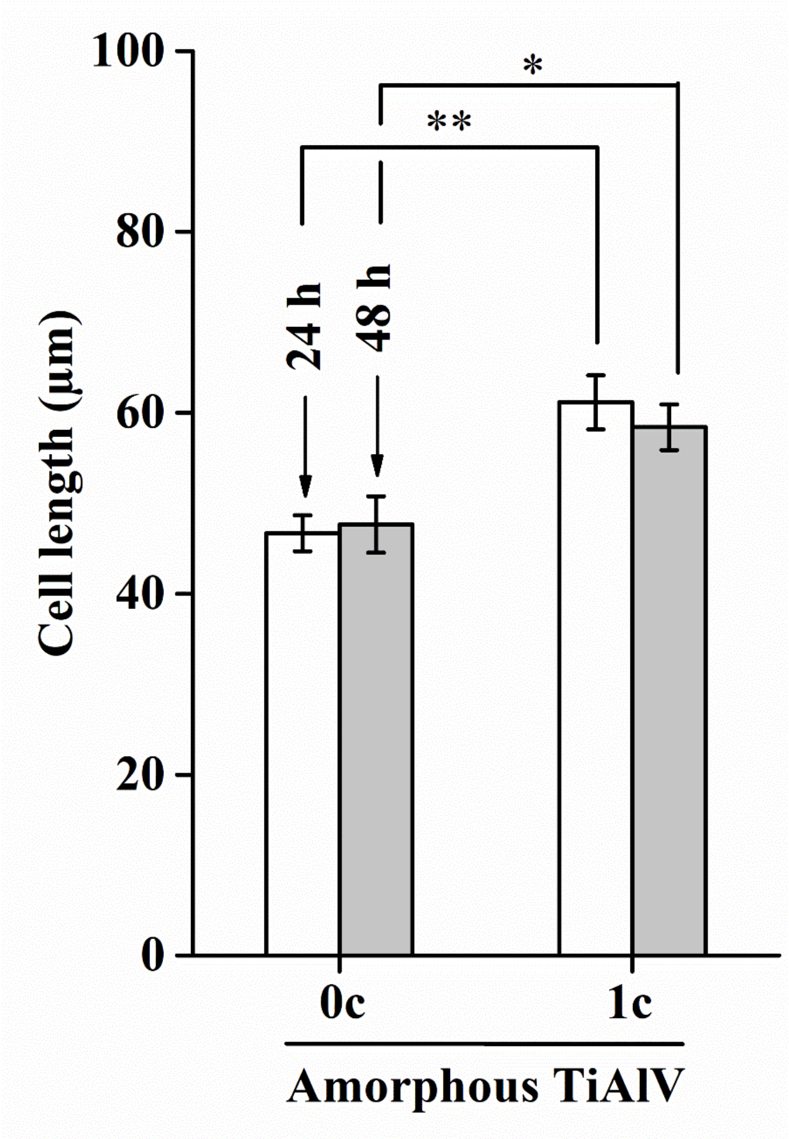

Figure S7. Analysis of elongation of MG-63 cells on uncoated and 1c TiO_2-coated AM-TiAlV foils grown for 24-72 h (AM = amorphous; 0c = without TiO_2 ALD coating; 1c TiO_2 = 1c TiO_2 ALD coating). The data are presented as mean ± SD *, $p < 0.05$; **, $p < 0.01$).

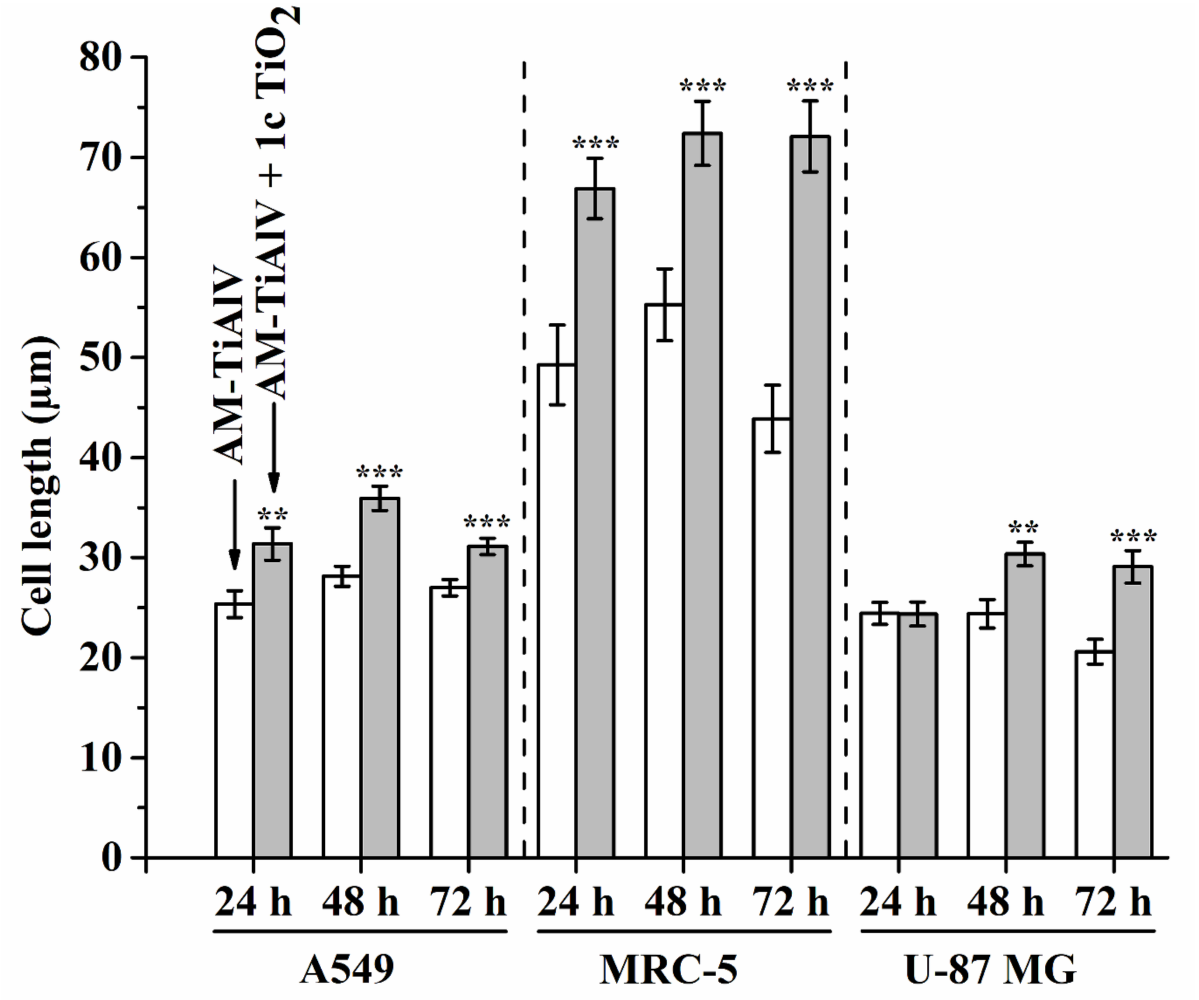

Figure S8. Analysis of elongation of A549, MRC-5 and U-87 MG cells on uncoated and 1c TiO_2-coated AM-TiAlV grown for 24-72 h (0c = without TiO_2 ALD coating; 1c = 1c TiO_2 ALD coating). The data are presented as mean ± SD (**, $p < 0.01$; ***, $p < 0.001$).

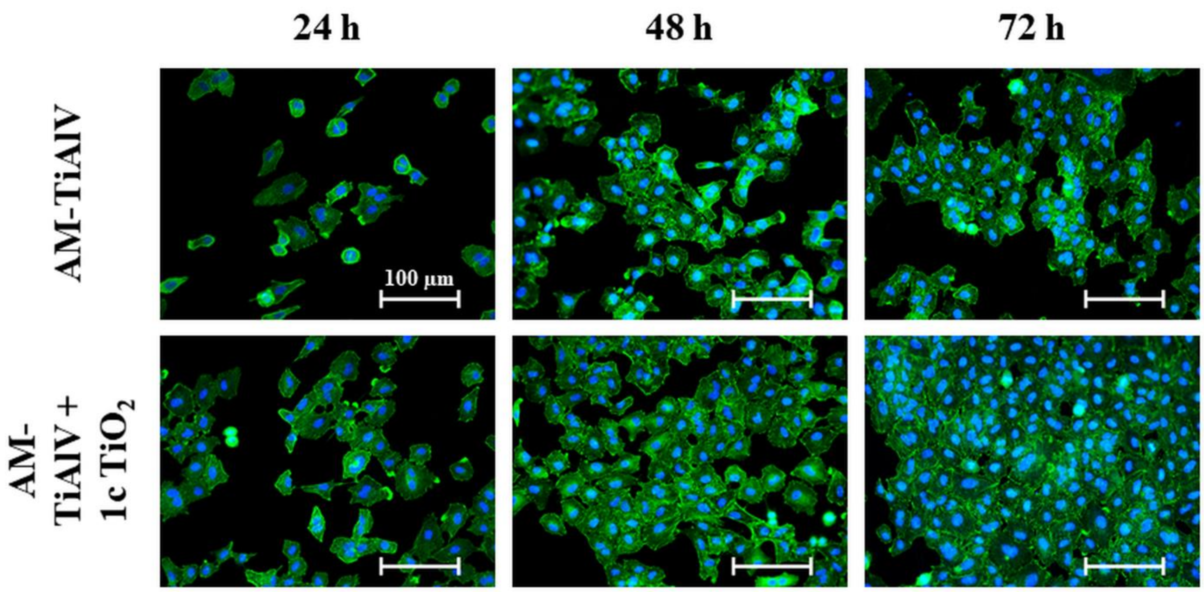

Figure S9. Photomicrographs of A549 cells on uncoated and 1c TiO_2-coated AM-TiAlV foils grown for 24-72 h. The actin filaments were stained with the Phalloidin-FITC probe (green), and cells' nuclei were stained with the Hoechst 33258 probe (blue).

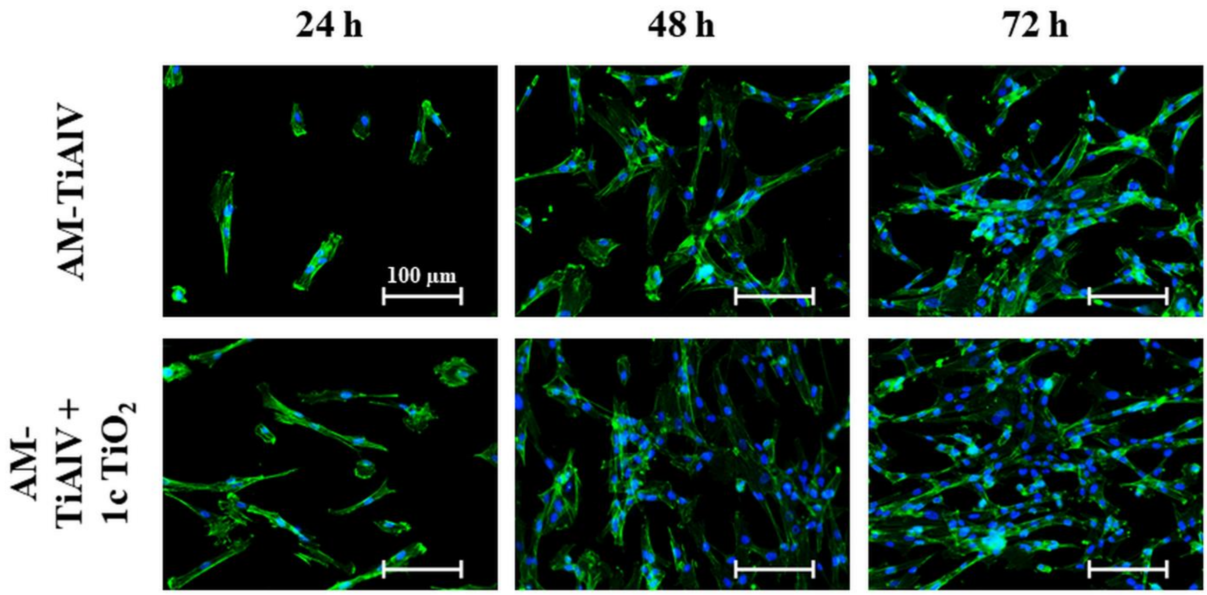

Figure S10. Photomicrographs of MRC-5 cells on uncoated and 1c TiO_2-coated AM-TiAlV foils grown for 24-72 h. The actin filaments were stained with the Phalloidin-FITC probe (green), and cells' nuclei were stained with the Hoechst 33258 probe (blue).

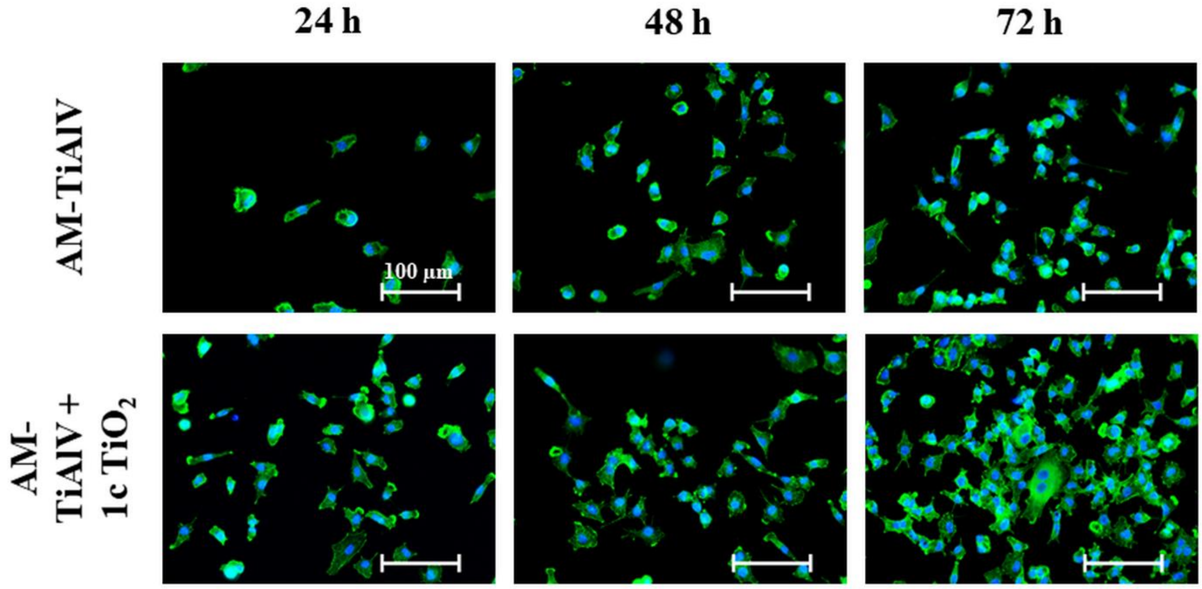

Figure S11. Photomicrographs of U-87 MG cells on uncoated and 1c TiO_2-coated AM-TiAlV foils grown for 24-72 h. Actin filaments were stained with the Phalloidin-FITC probe (green), and cells' nuclei were stained with the Hoechst 33258 probe (blue).

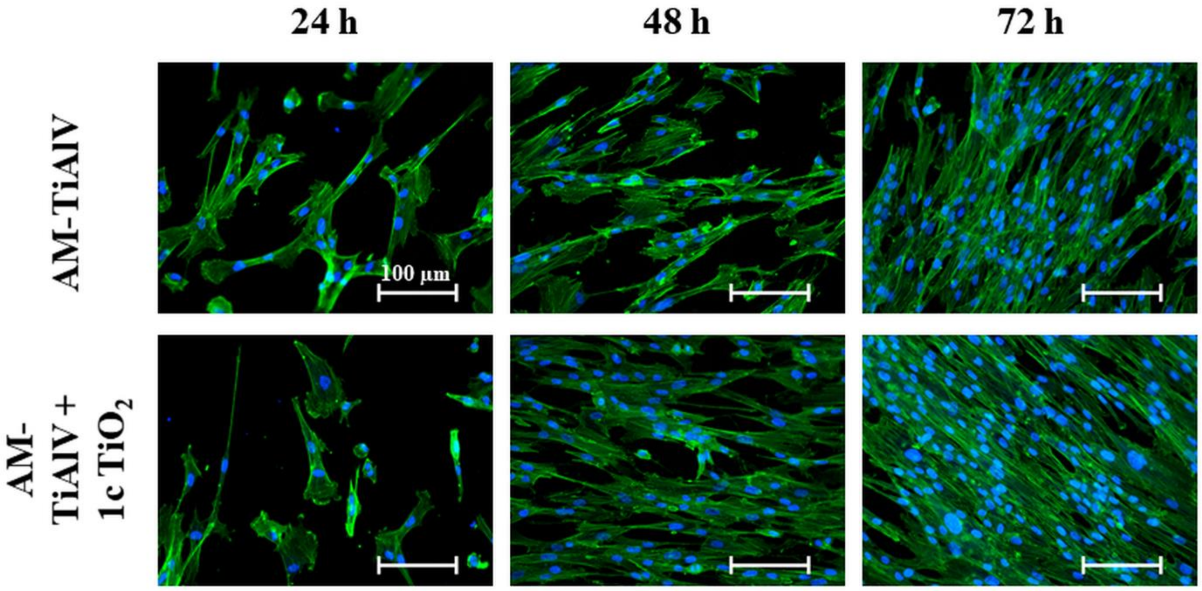

Figure S12. Photomicrographs of WI-38 cells on uncoated and 1c TiO_2-coated AM-TiAlV foils grown for 24-72 h. The actin filaments were stained with the Phalloidin-FITC probe (green), and cells' nuclei were stained with the Hoechst 33258 probe (blue).
